# Incongruent range dynamics between co‐occurring Asian temperate tree species facilitated by life history traits

**DOI:** 10.1002/ece3.2014

**Published:** 2016-03-06

**Authors:** Yun‐Peng Zhao, Xiao‐Ling Yan, Graham Muir, Qiong‐Yan Dai, Marcus A. Koch, Cheng‐Xin Fu

**Affiliations:** ^1^ The Key Laboratory of Conservation Biology for Endangered Wildlife of the Ministry of EducationCollege of Life SciencesZhejiang UniversityHangzhou310058China; ^2^ Laboratory of Systematic and Evolutionary Botany and BiodiversityInstitute of Ecology and Conservation Centre for Gene Resources of Endangered WildlifeZhejiang UniversityHangzhou310058China; ^3^ Shanghai Chenshan Plant Science Research CentreChinese Academy of SciencesShanghai Chenshan Botanical GardenShanghai201602China; ^4^ Department of Biodiversity and Plant SystematicsCentre for Organismal StudiesUniversity of HeidelbergIm Neuenheimer Feld 34569120HeidelbergGermany

**Keywords:** Asymmetric gene flow, *F*_ST_, *Ginkgo biloba*, late‐Pleistocene divergence, lifetime reproductive success, random genetic drift, temporal dynamics

## Abstract

Postglacial expansion to former range limits varies substantially among species of temperate deciduous forests in eastern Asia. Isolation hypotheses (with or without gene flow) have been proposed to explain this variance, but they ignore detailed population dynamics spanning geological time and neglect the role of life history traits. Using population genetics to uncover these dynamics across their Asian range, we infer processes that formed the disjunct distributions of *Ginkgo biloba* and the co‐occurring *Cercidiphyllum japonicum* (published data). Phylogenetic, coalescent, and comparative data suggest that *Ginkgo* population structure is regional, dichotomous (to west–east refugia), and formed *˜*51 kya, resulting from random genetic drift during the last glaciation. This split is far younger than the north–south population structure of *Cercidiphyllum* (~1.89 Mya). Significant (recent) unidirectional gene flow has not homogenized the two *Ginkgo* refugia, despite 2*Nm* > 1. Prior to this split, gene flow was potentially higher, resulting in conflicting support for a priori hypotheses that view isolation as an explanation for the variation in postglacial range limits. Isolation hypotheses (with or without gene flow) are thus not necessarily mutually exclusive due to temporal variation of gene flow and genetic drift. In comparison with *Cercidiphyllum*, the restricted range of *Ginkgo* has been facilitated by uncompetitive life history traits associated with seed ecology, highlighting the importance of both demography and lifetime reproductive success when interpreting range shifts.

## Introduction

The warm‐temperate deciduous forests of eastern Asia harbor a rich plant species diversity including many “living fossils” belonging to the Tertiary flora ~65.5–2.6 million years ago (Ma) (Axelrod et al. 1998; Manchester et al. 2009). The greatest concentration of these relicts (e.g., *Ginkgo*,* Metasequoia*,* Cercidiphyllum*,* Davidia*) presently extends from southwest China eastward across the Yangtze Valley to the east coast and as far as southern Japan (Lu 1999). These floral elements are considered to be relatively evolutionary stable because although affected by climate change, they were not directly affected by the ice sheets from continental glaciation. The region is thus associated with a reduced magnitude of Quaternary environmental change (≤2.6 Ma) compared with other temperate regions in the Northern Hemisphere (Liu 1988). The most recent paleobiome reconstructions, however, suggest rather more complex range dynamics for this forest biome during the Last Glacial Maximum (LGM; *˜*18,000–24,000 ya) as habitat loss (and gain) in different areas of temperate and subtropical China is thought to have occurred (Harrison et al. 2001; Ni et al. 2014). Thus, the genetic potential to detect population structure, population expansion/contraction may be rather higher than the absence of continental glaciation would suggest (reviewed in Qiu et al. 2011).

Consistent with the former view of limited Quaternary environmental change, molecular evidence from *Ginkgo biloba* suggests that natural populations have persisted in southwestern and eastern China, without exhibiting any strong genetic signatures of range expansion/contraction (Gong et al. 2008). Similar results in understory herbs also implicitly favor a “stable‐range” hypothesis, especially in the southern Yangtze Valley (Qiu et al. 2009a,b,c). However, a case study in *Cercidiphyllum japonicum*, another tree species inhabiting warm‐temperate deciduous Asian forests, suggests massive habitat losses in north‐central China but, conversely, increases in southwestern/eastern China during the late‐Pleistocene (Qi et al. 2012).

Two existing competing hypotheses (and a third presented here) may explain the variation in the degree of postglacial expansion to former range limits between temperate deciduous forest plants in eastern Asia. Qian and Ricklefs (2000) hypothesized that during glacial periods, isolated populations admixed/merged at lower elevations (isolation with admixture). Harrison et al. (2001), on the other hand, argued that they remained isolated during glacial as well as interglacial periods (continual isolation). The essential difference between the two hypotheses is one of admixture (negating isolation) during glacial periods. However, as several authors have noted, isolation is relative and the temporal dynamics of population gene flow and random genetic drift during the Pleistocene make the two hypotheses far from mutually exclusive. Thus, the time frame during which population genetic structure may have formed via drift and the pre‐/postdivergence dynamics of gene flow are critical in testing both hypotheses as is the use of multiple phylogeographical study systems to form robust independent interpretations of the two hypotheses. A third, albeit less considered, hypothesis is that life history traits can impose restrictions on the range dynamics and population genetic structure of temperate plants by their influence on lifetime reproductive success (Hamrick and Godt 1996).

The range dynamics of *Gingko biloba* and *Cercidiphyllum japonicum*, two wind‐pollinated dioecious trees, offer a means of testing the same set of hypotheses of genetic isolation and the possible roles of life history traits with data gathered from codistributed species. Both trees are flagship species of warm‐temperate forests in eastern Asia, co‐occurring in habitats with rocky outcrops in proximity to streams subject to frequent flooding disturbance (Tang et al. 2012), and their co‐occurrence has been conserved since the late Cretaceous (*c*. 66 Ma; Royer et al. 2003). The two species occupy similar climatic niches although the historical distribution of *C. japonicum* was possibly more northerly, as indicated by introgression with the cool‐temperate sister species *C. magnificum* (Qi et al. 2012). Both *G. biloba* and *C. japonicum* possess genetically diverged lineages which may have resulted from vegetation shifts and habitat displacement by the invasion of boreal forests during glacial periods. However, only *C. japonicum* shows the genetic signal of range expansion (Qi et al. 2012). Moreover, the small, flattened, winged seeds of *Cercidiphyllum* are dispersed by wind (Fu and Endress 2001), while the large fleshy seeds of *Ginkgo* are mostly dispersed by animals (Del Tredici 2007); a difference in seed dispersal which may lead to different outcomes during recolonization. The comparison of range dynamics between *Ginkgo* and *Cercidiphyllum* thus offers an ideal system to test the aforementioned set of hypotheses regarding gene flow and genetic isolation.

Using phylogenetic, coalescent, and comparative methods, we infer the processes responsible for the formation of disjunct distributions in these two species (new *Ginkgo* data and published *Cercidiphyllum* data; Qi et al. 2012), simultaneously assessing the degree of congruence among other members of the same ecosystem. Across its complete Asian range, we report the population genetic structure, the split time for divergent populations, and the rates of population migration for *Ginkgo*. From the split time, we aim to test whether *Ginkgo* lineages diverged in a congruent time frame as its floral associate, *Cercidiphyllum* (1.89 Ma). We date the *Ginkgo* split to a narrow window during glacial periods and compare the degree of genetic isolation experienced by the two species during these episodes. The effects of gene flow and several life history traits on the observed genetic structure are discussed, as are the degree of postglacial expansion to former range limits in eastern Asia and the variance in expansion observed among temperate tree taxa.

## Materials and Methods

### Tree samples

**Figure 1:**
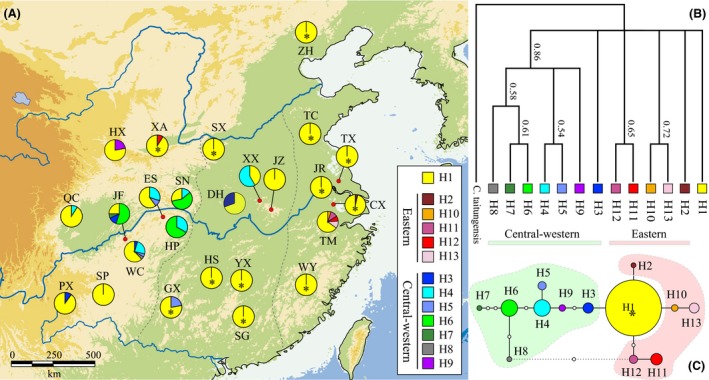
Geographical distribution and genealogical relationships (A) of 13 chloroplast (cp)DNA haplotypes detected in 25 *Ginkgo biloba* east Asian populations. Gray dash lines delineate (approximately) three geographical regions in China: western, central, and eastern. Population abbreviations correspond to those in Table S5 in Appendix S1. Asterisks indicate cultivated populations. Bayesian reconstruction of phylogenetic relationships (B) among cpDNA haplotypes using *Cycas taitungensis* as the outgroup (posterior probabilities are indicated for branches with >50% support). A statistical parsimony network is shown immediately below (C). In the latter, pie size is proportional to haplotype frequency, open circles represent missing haplotypes, and the asterisk indicates the most ancestral haplotype (with the highest probability). The dotted line indicates a potentially closed haplotype “loop.” Note, however, that haplotypes H12 and H8, respectively, connect to H1 and H6 with high certainty rather than to the missing haplotypes (see text).

Young leaves from a total of 761 individuals of *G. biloba* were collected from 25 populations in China (Fig. 1A; Table S1 in Appendix S1) and preserved in silica gel. This total includes 126 individuals from 13 populations analyzed previously (Gong et al. 2008). To exclude recent planting by humans, we sampled trees only with a diameter at breast height (DBH) of larger than 50 cm which corresponds to a minimum age of ~125 years (Tang et al. 2012). As far as possible, the populations sampled were located in or near natural habitats, confirmed using additional field investigations or reference to historical literature. Twelve presumably cultivated populations (XA, SX, ZH, TC, TX, JR, GX, CX, WY, YX, SG, HS) were included as controls, many of which have been genetically characterized with chloroplast markers (Shen et al. 2005; Gong et al. 2008). The latter studies suggest that any human‐mediated gene flow (assisted planting) is usually local and would not result in dichotomous genetic structure at a countrywide level.

### Molecular protocols

Genomic DNA was extracted from ~100 mg of silica gel‐dried leaf material using a modified CTAB protocol from Doyle and Doyle (1987). Three chloroplast DNA (cpDNA) regions were amplified in 354 individuals using the following primers: *trn*K1 and *trn*K2 for the *trn*K intron (Demensure et al. 1995), *trn*S and *trn*G for the *trn*S‐*trn*G intergenic spacer (IGS) (Hamilton 1999), and *atp*H and *atp*I for the *atp*H‐*atp*I IGS (Grivet et al. 2001). We included previously published *trn*K and *trn*S‐*trn*G regions for 124 individuals (Gong et al. 2008). PCR amplification was performed in a GeneAmp 9700 PCR (Applied Biosystems, Foster City, CA) using a total volume of 50 *μ*L containing 3 *μ*L 10× PCR buffer, 2 mmol/L MgCl_2_, 0.2 *μ*mol/L of each dNTP, 0.2 *μ*mol/L of each primer, 1.25 U *Taq* polymerase (Takara, Dalian, Liaoning, China), and 50 ng genomic DNA. Cycling conditions were 94°C for 5 min, followed by 35 cycles of 94°C for 1 min, annealing for 1 min (62°C for *trn*K, 58°C for *trn*S–*trn*G, and 52°C for *atp*H–*atp*I), and 72°C for 1.5 min (with a final extension step of 72°C for 10 min). Amplicons were purified using PCR Clean‐Up Kit (Bio‐engineering, Shanghai, China). Both strands were sequenced using *Taq* dye deoxy terminator cycle sequencing kit on an ABI 377XL DNA Sequencer (Applied Biosystems). Sequence alignment was performed using GENEIOUS 4.8 (Drummond et al. 2009) with visual inspection and manual adjustment. Unique haplotype sequences were deposited in GenBank (accession numbers of newly sequenced samples: KF220583–KF220586; and retrieved: EF468632–EF468642) (Table S2 in Appendix S1).

All 761 individuals were genotyped at 14 nuclear microsatellite (nSSR) loci (Yan et al. 2006, 2009) (Table S3 in Appendix S1). PCR amplification was performed in a reaction volume of 10 *μ*L containing 1 *μ*L of 10× PCR buffer, 1 mmol/L dNTPs, 0.5 *μ*mol/L each primer, 0.5 U of *Taq* polymerase, and 30–50 ng genomic DNA. The forward primers were labeled with a fluorescent dye (6‐FAM or HEX; Applied Biosystems). Amplification conditions were as follows: 94°C for 5 min followed by 35 cycles of 94°C for 1 min, the optimized annealing temperature (Table S3 in Appendix S1) for 30 sec, and 72°C for 45 sec (with a final extension step of 72°C for 7 min). Fragment analyses were performed on a MegaBACE 1000 autosequencer (GE Healthcare Bioscien‐ces, Pittsburgh, PA). To reduce scoring errors, allele sizes were manually scored twice with reference to an internal size standard (ET‐ROX 400) using Genetic Profiler 2.2 (GE Healthcare Biosciences).

### Plastid DNA sequence analyses

Each indel and inversion was treated as a single mutation, and coded as a nucleotide substitution, after Caicedo and Schaal (2004). Plastid DNA haplotypes were identified using DnaSP 5.10 (Librado and Rozas 2009) and unique haplotypes from the three cpDNA regions differed by at least one mutation from other sequence variants (Table S4 in Appendix S1). Haplotype diversity (*h*) and nucleotide diversity (*π*) were estimated for each population (*h*_S_, *π*_S_) and at the species levels (*h*_T_, *π*_T_) using the same program.

To quantify the proportion of total genetic variance explained by differences between regional groups of populations, we applied analyses of molecular variance (AMOVAs) based on Tamura and Nei's distance (1993) with 10,000 permutations using ARLEQUIN 3.5 (Excoffier and Lischer 2010).

Phylogenetic relationships among cpDNA haplotypes were reconstructed using Bayesian inference implemented in MrBayes 3.1.2 (Ronquist and Huelsenbeck 2003) with *Cycas taitungensis* as the outgroup (Wu et al. 2013; GenBank accession No. NC_009618). The best fitting substitution model, HKY + G, was determined according to Akaike Information Criterion (AIC) using jModelTest 0.1 (Posada 2008). Three independent runs of Metropolis‐coupled Markov chain Monte Carlo (MCMC) analysis were executed, each including one cold chain and three incrementally heated chains that started randomly in the parameter space. Each run included ten million generations with tree sampling every 1000 generations and a burn‐in of the first 20% of sampled trees. The convergence of chains was checked using Tracer 1.5 (Rambaut and Drummond 2009). The remaining trees were pooled to estimate the posterior probabilities. Genealogical relationships were also inferred by constructing a statistical parsimony network using TCS 1.21 (Clement et al. 2000). Because indels may be more prone to homoplasy (Kelchner 2000), we performed separate analyses by including and excluding indel sequences. The resulting network patterns were congruent.

### Microsatellite analyses

Linkage disequilibrium and Hardy–Weinberg equilibrium (10,000 dememorization steps, 100 batches, and 5000 iterations) were tested for each locus–population combination using GENEPOP 4.2 (http://www.genepop.curtin.edu.au/). Significance levels were adjusted using the sequential Bonferroni correction for multiple comparisons (Rice 1989). Genetic diversity, as measured by the observed number of alleles (*N*_A_), allele richness (*A*_R_), expected heterozygosity (*H*_E_), and observed heterozygosity (*H*_O_), was estimated per locus and per population using FSTAT 2.9.3 (Goudet 2001). Rare allelic richness (*R*_A_) was manually calculated following Hartl and Clark (1997).

Genetic structure was examined in both cpDNA and nSSR data sets using AMOVA based on the sum of squared size differences (Michalakis and Excoffier 1996) performed in ARLEQUIN 3.5. Genetic structure was further identified in the nSSRs using the Bayesian MCMC programs STRUCTURE 2.3.3 (Pritchard et al. 2000) and BAPS 6.0 (Cheng et al. 2013). The most likely number of clusters (*K*) was inferred using Evanno's Δ*K* method (Evanno et al. 2005) implemented in Structure Harvester Web v0.6.93 (Earl and VonHoldt 2012). The STRUCTURE assignment of individuals to *K* clusters was based on probabilities computed from genetic data and without prior information on sample location. To avoid detecting local optima, ten independent runs for each *K* value were performed, assuming the admixture model with correlated allele frequencies and using burn‐in and MCMC lengths of 100,000 and 1,000,000 iterations, respectively. The BAPS analysis was conducted to incorporate geographical information using prior information on spatial coordinates via the spatial clustering module. STRUCTURE and BAPS vary in their approaches to estimating distinct gene pools and how much ancestry individuals draw from each pool. STRUCTURE infers the highest likelihood for each gene pool and the admixture of genotypes using allele frequency and linkage disequilibrium information from the data set directly. BAPS, on the other hand, first infers the most likely number of gene pools in the sample population and then performs the most likely admixture of genotypes (Corander et al. 2004). This approach has more power in identifying hidden structure within populations (Corander and Marttinen 2006).

### Demographic parameters associated with regional populations

Two regional populations were defined according to (1) the genetic clusters revealed in BAPS and (2) the phylogeography of cpDNA haplotypes (see results). The twelve known cultivated populations were excluded from this analysis. *F*_ST_ between these two groups was calculated for each cpDNA locus using DnaSP and for each nSSR locus using Microsatellite Analyzer (MSA) 4.0.5 (Dieringer and Schlötterer 2003).

We used an isolation‐with‐migration (IM) model (Nielsen and Wakeley 2001), implemented in IMa2 (Hey 2010) to estimate the population parameters associated with a split model in which two daughter populations (of size *θ*_W_ and *θ*_E_) diverge from a common ancestor (of size *θ*_A_) at time *t* and continue to exchange migrants at rates *m*_0‐1_ and *m*_1‐0_. As the complete data set was too large to analyze using an MCMC approach, we generated a smaller subset consisting of 25 individuals from each regional population (by random sampling of both the nuclear and the chloroplast data sets). IMa2 was also used to conduct likelihood ratio tests of nested models to test whether models with unidirectional gene flow are a significantly better fit to the data than models with/without bidirectional gene flow by the method of Nielsen and Wakeley (2001).

After exploratory runs, the priors were as follows: population size parameters (*q*_0_, *q*_1_, and *q*_2_) = 100, split time *t* = 1, and migration (*m*_0_ and *m*_1_) = 10. We adopted an average mutation rate for cpDNA of 2.70 × 10^−10^ substitutions per site per year (s/s/yr) for cycads (Crisp and Cook 2011), which is similar to those for *Cercidiphyllum japonicum* (3.18 × 10^−10^ s/s/yr; Qi et al. 2012) and *Pinus* (2.2 × 10^−10^–4.2 × 10^−10^ s/s/yr; Willyard et al. 2007). Note that to accommodate the uncertainty in mutation rates, we allowed for relaxed intervals, 2.2 × 10^−10^–1.7 × 10^−9^ (Graur and Li 2000). Mutation rate priors for nSSR were derived from *Thuja plicata* (6.3 × 10^−4^, range 3.0 × 10^−5^–4.0 × 10^−3^ mutations per locus per generation; O'Connell and Ritland 2004). One hundred geometrically heated chains (using the heating parameters *h*_a_ = 0.99, *h*_b_ = 0.75) were run for 2,500,000 steps beyond a 500,000‐step burn‐in, and trees were sampled every 100 steps. Four runs with different seed numbers were implemented independently.

Because results from each replicate were similar, 10^5^ trees were concatenated into a single run in load‐tree mode and the “test nested models” option was activated. This option evaluates the likelihood of 24 models simpler than the full IM model by constraining parameters (other than divergence time) and rejecting those that are significantly lower scored than the full model based on a likelihood ratio test. To convert the parameter estimates to demographic units, we assumed a generation time of 160 years for *Ginkgo*. This is in line with the average age (as a proxy for generation time) of one refugial population (from which our population JF was sampled) based on a direct measurement of increment cores of 46 *Ginkgo* trees with DBH greater than 5 cm (Tang et al. 2012). Note that simple demographic calculations of generation time might bias the timing estimation and we apply it here with some caution. This generation time is comparable to the mean age (~200 years, DBH > 10 cm) of another temperate tree, *Quercus robur*, again using age as a surrogate for generation time (Ranius et al. 2009). Increasing the generation time to 210 years (the modal age of population JF) had negligible effects on parameter estimation (data not shown).

### Tests for departures from neutrality

ARLEQUIN was used to perform two neutrality tests: Tajima's *D* (Tajima 1989) and Fu's *F*_s_ (Fu 1997). Critical values for these statistics were obtained using 100,000 coalescent simulations. Mismatch distribution analyses (MDAs) were conducted using ARLEQUIN, and a goodness‐of‐fit based on the sum of squared deviations (*SSD*) and Harpending's (1994) raggedness index (*H*_Rag_) was tested using parametric bootstrapping (Schneider and Excoffier 1999) with 1000 replicates.

## Results

### Rooting all chloroplast polymorphisms reveals H1 as the ancestral haplotype

The reconstructed Bayesian tree of 13 cpDNA haplotype sequences indicates the monophyly of all the observed haplotypes from central and western China (except H1 and H3) (86% Bayesian posterior support, Fig. 1B). The remaining haplotypes are not grouped into one single clade. The parsimony network (Fig. 1C) provided better resolution, although the network contained a potentially closed loop. This ambiguity can be easily resolved, however, as the haplotype in question (H12) connects with high certainty to the co‐occurring H11 haplotype rather than to the alternative H8 haplotype from a remote population (Crandall and Templeton 1993). The widespread haplotype H1 was inferred to be the most ancestral, giving rise to two groups of haplotypes (Fig. 1C).

Chloroplast sequences also indicate that more than half of the analyzed populations (14/25) are polymorphic (Table S5; Fig. 1A) with the majority occurring in the west (9/10 populations). Central and eastern populations were significantly less polymorphic (*P *<* *0.05, Wilcoxon sign test) compared to western ones with only 3/8 and 2/7 populations being polymorphic, respectively. Nine of the twelve known cultivated populations were monomorphic (except XA, GX, and CX) and fixed with the widespread haplotype H1.

### Maternally and biparentally inherited markers exhibit strong west–east population structure

The majority of derived cpDNA haplotypes show west–east geographical divisions (Fig. 1; Table 1). Haplotypes H3–H9, for example, are specific to central and western China, while H2 and H10–H13 are distributed in eastern China (one exception; H12 does occur in the western cultivated population XA, however). The widespread ancestral haplotype H1 is present in nearly all sampled populations across *Ginkgo*'s natural range.

**Table 1:** Analyses of molecular variance (AMOVAs) of hierarchical population structure in regional *G. biloba* populations in China

	cpDNA			nSSR		
Source of variation	df	Percentage of variation (%)	Ф^a^	df	Percentage of variation (%)	*R*‐statistics
Between West–East	1	16.64	Ф_CT_ = 0.166***	1	2.54	*R*_CT_ = 0.025***
Among populations within West–East	11	30.72	Ф_SC_ = 0.369***	11	9.42	*R*_SC_ = 0.094***
Within populations	203	52.64	Ф_ST_ = 0.474***	1173	88.04	*R*_ST_ = 0.120***

a Φ is a fixation index similar to Wright's *F*‐statistics. It reflects the correlation of random pairs of haplotypes drawn from a group relative to the correlation of pairs of random haplotypes drawn from the whole data set (Excoffier et al. 1992). Significance is indicated as follows: ***P <* *0.001.

**Figure 2:**
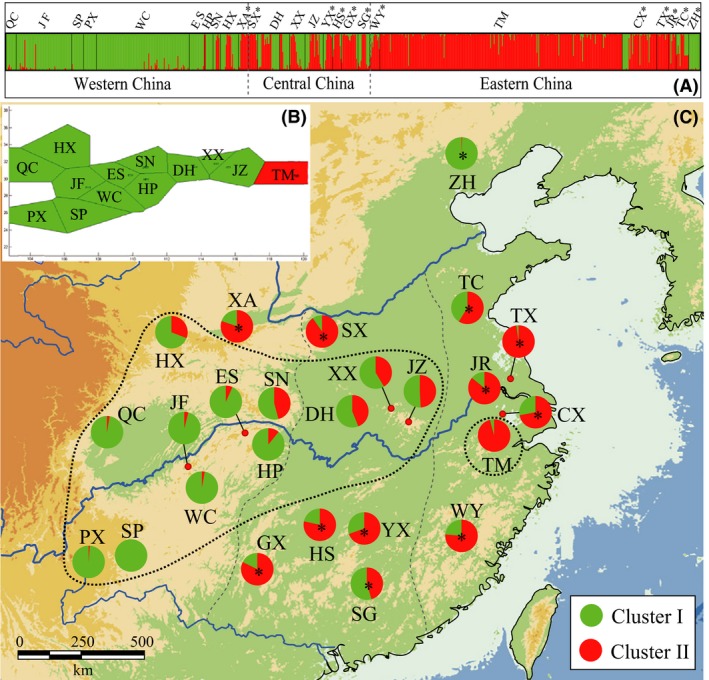
STRUCTURE (A) and BAPS (B) analyses of 25 populations (761 individuals) of *Ginkgo biloba* based on 14 nuclear microsatellite (nSSR) loci. Cultivated populations were excluded in spatial clustering using BAPS. Vertical bars in the STRUCTURE assignment plot represent individuals and admixture proportions for each one of two (*K *=* *2) genetic clusters. Geographical distribution of nSSR clusters (C). Black dotted lines indicate demarcation of the two genetic clusters (“WEST” and “EAST”) defined using BAPS (see text). Gray dash lines approximate three geographical regions delineated in China: western, central, and eastern. Population abbreviations correspond to those in Table S5 in Appendix S1. Asterisks indicate cultivated populations.

Bayesian‐based mixture analysis of all populations (based on the nSSR data) using prior information on spatial coordinates (BAPS) revealed that the best genetic partition within the data had a clear west–east spatial component (Fig. 2B), supporting the dichotomous west–east division inferred from both the haplotype network and the AMOVA.

Similarly, the STRUCTURE analysis revealed that Δ*K* peaked at *K* = 2 (Fig. S1 in Appendix S1). Individuals from western China (QC, JF, PX, SP, WC, ES, HP) were assigned to one cluster (“green”), while those from the eastern population (TM) were assigned to a different cluster (“red”) (Fig. 2A,C). Individuals from central China were variously assigned to one or other of these two genetic clusters.

AMOVA suggests that genetic variation is significantly structured at the regional (west/east) level for both the maternally inherited cpDNA (Ф_CT_ = 0.166, *P *<* *0.001) and biparentally inherited nSSR (*R*_CT_ = 0.025, *P *<* *0.001; Table 1). Within either the “west” or “east” group, however, most of the variation is partitioned within populations (~53% cpDNA and ~88% nSSR, respectively), with a significant component of variation among populations in these two regions.

### Recent population split and asymmetrical gene flow

**Figure 3:**
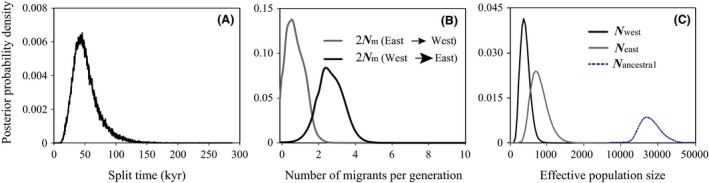
Marginal posterior probability distributions for the demographic parameters estimated under the isolation‐with‐migration model (Hey 2010). Time (A) since daughter populations split from a common ancestor is 51,355 (17,103–95,000) years. Population migration rates (B) are WEST→EAST 2.502 (0.456–4.525), while the value in the opposite direction is 0.933 (0–1.889). The latter migration rate does not significantly differ from zero, as suggested by LLR tests. Effective population sizes (C) are shown for group “WEST” (black): 416 (95% HPD: 156–870); group “EAST” (gray): 802 (335–1406); and “ancestral” population (dashed): 27,225 (15,555–40,282).

We fitted an isolation‐with‐migration model to the west/east population structure apparent within *Ginkgo* in order to estimate the split time from a common ancestor for these two regional groups. The estimated divergence time for the split between the two groups identified in the BAPS and STRUCTURE analyses was as recent as 51,355 years ago (95% HPD range: 17,103–95,000) (Fig. 3). Interestingly, following the split, the two regions have exchanged genes asymmetrically. Gene flow from west to east was 2.502 population migrants per generation (0.456–4.525), while gene flow in the converse direction was not statistically significant, 0.889 per generation (0–1.889). A model which assumes unidirectional gene flow between the two regions (from west to east) was a significantly better fit than models without gene flow or gene flow in the opposite direction (from east to west, Table S6 in Appendix S1).

### No footprint of a genetic bottleneck

From the IMa2 analysis, we also inferred extremely small effective population sizes for the derived (west and east) daughter populations (in the order of 100's), which is an order of magnitude smaller than their census sizes. The magnitude of these effective population sizes may be indicative of demographic changes and thus genetic bottlenecks. The latter, for example, may have resulted in the loss of (rare) genetic variants; detectable in a skew in allele frequency spectra. Nevertheless, *G. biloba* shows comparatively high levels of both cpDNA and nSSR diversity, which vary minimally among natural populations (Table S5 in Appendix S1). Analysis of the nucleotide frequency spectra in cpDNA indicated no significant signal of population expansion; Tajima's *D* was slightly negative but not significantly so (Table S7 in Appendix S1).

## Discussion

By comparing population genetic data from *Ginkgo biloba* with our (in‐house) published results of *Cercidiphyllum japonicum* (Qi et al. 2012) together with published Asian case studies, we provide some robust interpretations below of the hypotheses concerning variation in the degree of postglacial expansion of warm‐temperate deciduous forests in eastern Asia. The detailed dynamic history gleaned from the population genetic analyses allows the investigation of a process that includes isolation‐with‐migration models, random genetic drift, and the split times of population genetic structure in the context of isolation hypotheses proposed for the range dynamics of flora during glacial and interglacial periods.

### Regional Ginkgo population structure results from genetic drift in the late‐Pleistocene

**Figure 4:**
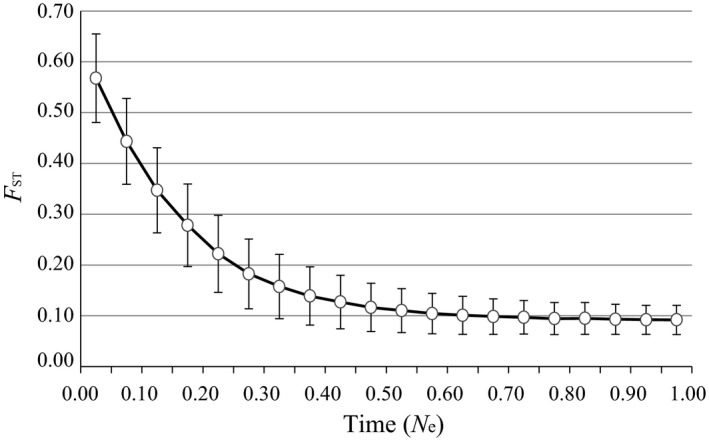
Population differentiation (*F*_ST_) between two subpopulations in an asymmetric island model with migration. Polymorphism data were generated by coalescent simulations in the *ms* program (Hudson 2002). The simulations assumed that all demes were of equal size with *θ* = 1.7, and 50 sequences were sampled from each subpopulation, respectively. One subpopulation received migrants at the same rate from the other subpopulation. A migration parameter (4*N*_0_ *m*) of 5.0 and 1.86 was assumed for subpopulation WEST and EAST, respectively. For each run, gene flow was introduced at time *T* (measured in *N*_e_) generations ago at intervals of 0.05*N*_e_. Dots represent the average differentiation (*F*_ST_; error bars represent standard deviations from 10,000 replicates) measured *T* generations after gene flow was introduced.

The IMa2 analysis suggests significant evidence for unidirectional gene flow from west to east. Nevertheless, significant population differentiation between west and east is observed for each chloroplast locus (*F*_ST_ = 0.148–0.301, *P *<* *0.001) and for each nSSR locus (0.002–0.081, *P *<* *0.01). Gene flow is expected to reduce or eliminate this differentiation. Low levels of migration (i.e., > one migrant per generation) are sufficient to prevent population differentiation by drift (Slatkin 1987). However, west–east population differentiation is apparent despite asymmetrical gene flow acting as a homogenizing force between these regions. Trees have long generation times, and together with the recent split, it may take many thousands of generations of gene flow to diminish this population structure. To illustrate this point, we ran coalescent simulations (in *ms*, Hudson 2002) for a two‐deme model with asymmetrical migration, using parameters estimated for *G. biloba* from our IM analysis (Fig. 3). From these simulations, at least 0.5*N*e generations of gene flow are required for *F*_ST_ to reach equilibrium (Fig. 4). The magnitude of simulated *F*_ST_ apparent between populations at 0.4*N*_e_ generations ago is comparable to the average *F*_ST_ (0.14) we observe from our cpDNA haplotype and nSSR data. This timing (0.4*N*_e_ generations) in turn corresponds to approximately 39,000 years ago (using an average estimate for *N*_e_ from western and eastern populations and a generation time of 160 years). The simulations suggest that the relatively high *F*_ST_ we observe between the regional populations dates from ~39 k years ago and may mirror the lag before *F*_ST_ reaches equilibrium between the lineage sorting (random genetic drift) of population divergence and the homogenizing effects of asymmetrical gene flow.

### Conflicting support for isolation hypotheses during glacial periods

While the present study confirms the presence of two genetically distinct populations associated with independent glacial refugia identified by Gong et al. (2008), one intention here was to shed light on the demography behind these west–east regional refugia and to identify where the population split (and associated genetic drift) lies in relation to geological time during the last glacial period. The recent split between the two populations at *˜*51 kya (95% HPD: 17,103–95,000; Fig. 3), fits firmly within the last glaciation (10–73 kya) in China (Yi et al. 2005). This suggests that the reduced range (and effective population size) of *G. biloba* dates, in part, from genetic drift in the late‐Pleistocene. Moreover, prior to the split, gene flow among populations across the range might have been higher, and/or the effective population size might have been larger in the historical migrant gene pool than observed after the split. These inferences are supported by Tajima's *D* values and the mismatch distribution, both of which did not reveal any signals of population expansion (Table S7 in Appendix S1). We may thus infer that the demography of *Ginkgo* includes recent fragmentation of a formerly larger population with probably long‐term in situ persistence (see also *Eurycorymbus cavaleriei*; Wang et al. 2009).

Assuming this inference of gene flow and/or a large effective population size prior to the split is correct, together with the asymmetrical gene flow observed following the split (Fig. 3B), it appears that west–east regional *Ginkgo* populations have not remained genetically isolated during the last glaciation. There is thus both evidence to support the Harrison et al. (2001) isolation hypothesis during glacial periods (i.e., sufficient isolation for drift to create the regional split) and logical inference to reject it, as there is genetic connectivity prior to the split, and recent asymmetrical gene flow between regions following the split.

This illustrates that isolation hypotheses (with or without gene flow) fail to account for the temporal dynamics of both isolation and gene flow among populations during glacial and interglacial periods. A model which includes isolation with migration is a good fit to the *Ginkgo* system and yet the coalescent simulations (for a two‐deme model with asymmetrical migration) confirm that genetic population structure (and apparent isolation) can persist in the presence of (recent) gene flow. Harrison et al. (2001) and Qian and Ricklefs' (2000) hypotheses suffer by not recognizing the potential for this temporal complexity. Only by unraveling population genetic processes such as those revealed in this study, are these subtleties revealed.

### Incongruence of demography between co‐occurring species may be facilitated by life history traits

The west–east dichotomous regional genetic structure that we observe in *Ginkgo* is also apparent in numerous other temperate woody plants from eastern Asia, for example, *Taxus wallichiana* (Gao et al. 2007), *Quercus variabilis* (Chen et al. 2012), and *Kalopanax septemlobus* (Sakaguchi et al. 2012). The genetic structure observed in *Cercidiphyllum* is approximately north–south (Fig. 1 in Qi et al. 2012). However, for most documented Asian temperate tree species, the division either lies between central‐west and eastern China/Asia (e.g., *Ginkgo*,* Q. variabilis*) or shifted clockwise along a northwest–southeast axis between Sino‐Himalayan and Sino‐Japanese forests (e.g., *T. wallichiana*,* K. septemlobus*). Among all of the taxa, the ages of these regional divisions varies substantially, from the youngest in *Ginkgo* ~51 kya to the oldest in *Cercidiphyllum* 1.89 Ma. In between these ranges, the genetic structure has been dated to 1.45 Ma in *Q. variabilis* and to 0.28–0.74 Ma in *K. septemlobus*. Interestingly, particularly for inferences regarding in situ persistence versus expansion hypotheses, postglacial expansions to former range limits differ among these species. Temporal and spatial incongruences suggest that these four trees did not respond to climate‐driven vegetation shifts in the same manner. One explanation for the incongruences may be differences in life history traits which we examine in detail below for two of the more ecologically associated taxa, *Gingko* and *Cercidiphyllum*.

Given the maternal inheritance of cpDNA in both *Ginkgo* and *Cercidiphyllum*, the range expansion observed in *Cercidiphyllum* following glaciation may imply comparatively poorer seed dispersal in *Ginkgo*. Indeed, the fleshy (heavy) seeds of *Ginkgo* are probably dispersed less efficiently than the wind‐dispersed seeds of *Cercidiphyllum*. Knowledge of the seed dispersers in *Ginkgo* has been speculative (Del Tredici 2007; Del Tredici 1992; Jiang et al., 1990) but some information about the effectiveness of seed dispersers can be inferred from the distribution of maternally inherited chloroplast haplotypes. With the exception of the widespread ancestral H1, no derived cpDNA haplotypes were shared between closely located populations, for example, TM and CX (<100 km apart), suggesting that *Ginkgo* seeds were not dispersed efficiently. Nevertheless, the widespread H1 and its direct relatives were shared between distantly located populations, suggesting that the commonality among geographically dispersed haplotypes results from shared ancestry rather than animal (or human) mediated gene flow. The split time (~51 ka) for the regional population structure far predates that of human‐mediated introductions (<2 ka), and is too recent to have allowed new mutations to generate five newly derived haplotypes specific to eastern China. We thus consider animal mediated gene flow via seeds to be limited.

*Ginkgo* is also at a comparative disadvantage to *Cercidiphyllum* in disturbed riparian rocky environments because its large seeds with slowly developing embryos require a level of stability less often available in habitats with frequent disturbance and flooding (Del Tredici 2007). Furthermore, the slow and complex sexual reproductive cycle in *Ginkgo* is highly constrained by temperature (Hatano and Kano 1952; Del Tredici 2007). As a result, sexual reproduction may have been inhibited during the last glaciation, resulting in range contraction. Limited seed dispersal, poor competition to recolonize riparian habitats, and inefficient sexual reproduction would have severely constrained the plant's ability to spread beyond refugia following range contraction. Of the two species, *Gingko* may have been more sensitive to climatic cooling and aridification at the beginning of the Pleistocene. Therefore, the influence of climatic niche differentiation in the demographic histories of these two species cannot be discounted.

On balance, seed dispersal coupled with the long generation time in *Ginkgo*, are important factors in explaining the presence of (restricted) regional *Ginkgo* populations in China. Life history traits can impose restrictions on the range dynamics and population genetic structure of temperate plants by their influence on lifetime reproductive success (Hamrick and Godt 1996) and should be considered as a valid alternative to previously proposed hypotheses to explain variation in the degree of postglacial expansion to former range limits between co‐occurring species of warm‐temperate deciduous forests.

## Conclusions

Two competing isolation hypotheses (with or without gene flow) have been proposed to explain the variance of postglacial expansion to former range limits among warm‐temperate deciduous Asian tree species. During glacial periods, isolation with admixture at lower altitudes (Qian and Ricklefs 2000), or continual isolation without admixture (Harrison et al. 2001; negating any isolation, if 2*Nm* > 1), may explain this variance. However, proving that isolation occurred continuously throughout a glacial period is challenging. Gene flow and random genetic drift can vary both temporally and spatially throughout glacial periods. Both isolation hypotheses are thus not necessarily mutually exclusive, and they also neglect the effect of life history traits on range dynamics and population genetic structure via their influence on lifetime reproductive success. Using population genetics to uncover the dynamism of postglacial history, this study illustrates the inadequacies of “isolation” hypotheses that span geological time where population dynamics are the norm. The comparison between *Ginkgo* and other taxa, particularly *Cercidiphyllum*, sampled across the same ecosystem indicates that co‐occurring species may not have congruent range dynamics. This highlights the importance of both demography and life history traits when interpreting range shifts and the evolution of ecological communities.

## Conflict of Interest

None declared.

## Supporting information


**Appendix S1.** Sampling information, GenBank accession numbers, population diversity of nSSRs and cpDNA haplotypes, tests of IM nested models, neutrality tests, and likelihood values of the cluster number in Bayesian assignment analyses.
**Table S1.** Details of sample locations, voucher number, latitude, longitude and altitude in 25 *G. biloba* populations.
**Table S2.** GenBank accession numbers of cpDNA variants.
**Table S3.** Locus, repeat motifs, size ranges, number of alleles (*N*_A_), observed (*H*_O_) and expected heterozygosity (*H*_E_) for fourteen microsatellite loci analyzed in 25 populations of *G. biloba*.
**Table S4.** Chloroplast DNA sequence polymorphisms detected in the three regions of *Ginkgo biloba* identify 13 haplotypes (H1–H13).
**Table S5.** Plastid and nuclear diversity in 25 *G. biloba* populations sampled across its complete natural range in China.
**Table S6.** Tests of nested models for the WEST versus EAST group for the three cpDNA loci and 14 nSSR loci data set.
**Table S7.** Summary of neutrality tests and mismatch distribution parameters for groups WEST and EAST of *G. biloba*. Estimates of mismatch distribution were obtained under models of spatial expansion using ARLEQUIN.
**Fig. S1** Likelihood values across multiple values of the cluster number in Bayesian assignment analyses, *K* (1–10) using Evanno's methods (Evanno et al. 2005).

## References

[ece32014-bib-0001] Axelrod, D. I. , I. Al‐Shehbaz , and P. H. Raven . 1998 History of the modern flora of China Pp. 43–55 *in* Zhang A. L. and Wu S. G., eds. Floristic Characteristics and Diversity of East Asian Plants. China Higher Education Press, Beijing.

[ece32014-bib-0002] Caicedo, A. L. , and B. A. Schaal . 2004 Population structure and phylogeography of *Solanum pimpinellifolium* inferred from a nuclear gene. Mol. Ecol. 13:1871–1882. 15189210 10.1111/j.1365-294X.2004.02191.x

[ece32014-bib-0003] Chen, D. , X. Zhang , H. Kang , X. Sun , S. Yin , H. Du , et al. 2012 Phylogeography of *Quercus variabilis* based on chloroplast DNA sequence in East Asia: multiple glacial refugia and mainland‐migrated island populations. PLoS ONE 7:e47268. 23115642 10.1371/journal.pone.0047268 PMC3480369

[ece32014-bib-0004] Cheng, L. , T. R. Connor , J. Sirén , D. M. Aanensen , and J. Corander . 2013 Hierarchical and spatially explicit clustering of DNA sequences with BAPS software. Mol. Biol. Evol. 30:1224–1228. 23408797 10.1093/molbev/mst028 PMC3670731

[ece32014-bib-0005] Clement, M. , D. Posada , and K. A. Crandall . 2000 TCS: a computer program to estimate gene genealogies. Mol. Ecol. 9:1657–1659. 11050560 10.1046/j.1365-294x.2000.01020.x

[ece32014-bib-0006] Corander, J. , and P. Marttinen . 2006 Bayesian identification of admixture events using multilocus molecular markers. Mol. Ecol. 10:2833–2843. 16911204 10.1111/j.1365-294X.2006.02994.x

[ece32014-bib-0007] Corander, J. , P. Waldmann , P. Marttinen , and M. J. Sillanpää . 2004 BAPS 2: enhanced possibilities for the analysis of genetic population structure. Bioinformatics 20:2363–2369. 15073024 10.1093/bioinformatics/bth250

[ece32014-bib-0008] Crandall, K. A. , and A. R. Templeton . 1993 Empirical tests of some predictions from coalescent theory with applications to intraspecific phylogeny reconstruction. Genetics 134:959–969. 8349118 10.1093/genetics/134.3.959 PMC1205530

[ece32014-bib-0009] Crisp, M. D. , and L. G. Cook . 2011 Cenozoic extinctions account for the low diversity of extant gymnosperms compared with angiosperms. New Phytol. 192:997–1009. 21895664 10.1111/j.1469-8137.2011.03862.x

[ece32014-bib-0010] Del Tredici, P. 2007 The phenology of sexual reproduction in *Ginkgo biloba*: ecological and evolutionary implications. Bot. Rev. 73:267–278.

[ece32014-bib-3000] Del Tredici, P. 1992 Natural regeneration of *Ginkgo biloba* from downward growing cotyledonary buds (Basal Chichi). Am. J. Bot. 79:522‐530.

[ece32014-bib-0011] Demensure, B. , N. Sodzi , and R. J. Petit . 1995 A set of universal primers for amplification of polymorphic noncoding regions of mitochondrial and chloroplast DNA in plants. Mol. Ecol. 4:129–131. 7711952 10.1111/j.1365-294x.1995.tb00201.x

[ece32014-bib-0012] Dieringer, D. , and C. Schlötterer . 2003 Microsatellite analyser (MSA): a platform independent analysis tool for large microsatellite data sets. Mol. Ecol. Notes 3:167–169.

[ece32014-bib-0013] Doyle, J. J. , and J. L. Doyle . 1987 A rapid DNA isolation procedure for small quantities of fresh leaf tissue. Phytochem. Bull. 19:11–15.

[ece32014-bib-0014] Drummond, A. J. , B. Ashton , M. Cheung , J. Heled , M. Kearse , R. Moir , et al. 2009 Geneious v4.8. http://www. geneious. com

[ece32014-bib-0015] Earl, D. , and B. VonHoldt . 2012 STRUCTURE HARVESTER: a website and program for visualizing STRUCTURE output and implementing the Evanno method. Conserv. Genet. Resour. 4:359–361.

[ece32014-bib-0016] Evanno, G. , S. Regnaut , and J. Goudet . 2005 Detecting the number of clusters of individuals using the software structure: a simulation study. Mol. Ecol. 14:2611–2620. 15969739 10.1111/j.1365-294X.2005.02553.x

[ece32014-bib-0017] Excoffier, L. , and H. E. L. Lischer . 2010 Arlequin suite ver 3.5: a new series of programs to perform population genetics analyses under Linux and Windows. Mol. Ecol. Resour. 10:564–567. 21565059 10.1111/j.1755-0998.2010.02847.x

[ece32014-bib-0018] Excoffier, L. , P. E. Smouse , and J. M. Quattro . 1992 Analysis of molecular variance inferred from metric distances among DNA haplotypes: application to human mitochondrial DNA restriction data. Genetics 131:479–491. 1644282 10.1093/genetics/131.2.479 PMC1205020

[ece32014-bib-0019] Fu, X. Y. 1997 Statistical tests of neutrality of mutations against population growth, hitchhiking and background selection. Genetics 147:915–925. 9335623 10.1093/genetics/147.2.915 PMC1208208

[ece32014-bib-0020] Fu, D. Z. , and P. K. Endress . 2001 Cercidiphyllaceae Pp. 126 *in* Wu Z. Y., Raven P. H., Hong D. Y., eds. Flora of China. Science Press, Beijing, China.

[ece32014-bib-0021] Gao, L. M. , M. Möller , X. M. Zhang , M. L. Hollingsworth , J. Liu , R. R. Mill , et al. 2007 High variation and strong phylogeographic pattern among cpDNA haplotypes in *Taxus wallichiana* (Taxaceae) in China and North Vietnam. Mol. Ecol. 16:4684–4698. 17908214 10.1111/j.1365-294X.2007.03537.x

[ece32014-bib-0022] Gong, W. , C. Chen , C. Dobes , C. X. Fu , and M. A. Koch . 2008 Phylogeography of a living fossil: pleistocene glaciations forced *Ginkgo biloba* L. (Ginkgoaceae) into two refuge areas in China with limited subsequent postglacial expansion. Mol. Phylogenet. Evol. 48:1094–1105. 18554931 10.1016/j.ympev.2008.05.003

[ece32014-bib-0023] Goudet, J. 2001 FSTAT, a program to estimate and test gene diversities and fixation indices, http://www.unil.ch/izea/softwares/fstat.htlm

[ece32014-bib-0024] Graur, D. , and W. H. Li . 2000 Fundamentals of molecular evolution. Sinauer Associates Inc., Publishers, Sunderland, MA.

[ece32014-bib-0025] Grivet, D. , B. Heinze , G. G. Vendramin , and R. J. Petit . 2001 Genome walking with consensus primers: application to the large single copy region of chloroplast DNA. Mol. Ecol. Notes 1:345–349.

[ece32014-bib-0026] Hamilton, B. M. 1999 Four primer pairs for the amplification of chloroplast intergenic regions with intraspecific variation. Mol. Ecol. 8:521–523. 10199016

[ece32014-bib-0027] Hamrick, J. L. , and M. J. Godt . 1996 Effects of life history traits on genetic diversity in plant species. Philos. Trans. R. Soc. Lond. B Biol. Sci. 351:1291–1298.

[ece32014-bib-0028] Harpending, H. C. 1994 Signature of ancient population growth in a low‐resolution mitochondrial DNA mismatch distribution. Hum. Biol. 66:591–600. 8088750

[ece32014-bib-0029] Harrison, S. P. , G. Yu , H. Takahara , and I. C. Prentice . 2001 Palaeovegetation (Communications arising): diversity of temperate plants in east Asia. Nature 413:129–130. 11557970 10.1038/35093166

[ece32014-bib-0030] Hartl, D. , and A. Clark . 1997 Principles of population genetics. Sinauer, Sunderland, MA.

[ece32014-bib-0031] Hatano, K. , and T. Kano . 1952 A brief report on the after‐ripening of the seeds of *Ginkgo biloba* . J. Jpn. For. Soc. 34:369–370.

[ece32014-bib-0032] Hey, J. 2010 Isolation with migration models for more than two populations. Mol. Biol. Evol. 27:905–920. 19955477 10.1093/molbev/msp296 PMC2877539

[ece32014-bib-0033] Hudson, R. R. 2002 Generating samples under a Wright‐Fisher neutral model of genetic variation. Bioinformatics 18:337–338. 11847089 10.1093/bioinformatics/18.2.337

[ece32014-bib-3001] Jiang, M. X. , Y. X. Jin , and Q. F. Zhang . 1990 A preliminary study on *Ginkgo biloba* in Dahongshan Region. Hubei. J. Wuhan Bot. Res. 8:191‐193.

[ece32014-bib-0034] Kelchner, S. A. 2000 The evolution of non‐coding chloroplast DNA and its application in plant systematics. Ann. Mo. Bot. Gard. 87:482–498.

[ece32014-bib-0035] Librado, P. , and J. Rozas . 2009 DnaSP v5: a software for comprehensive analysis of DNA polymorphism data. Bioinformatics 25:1451. 19346325 10.1093/bioinformatics/btp187

[ece32014-bib-0036] Liu, K. B. 1988 Quaternary history of the temperate forests of China. Quatern. Sci. Rev. 7:1–20.

[ece32014-bib-0037] Lu, A. M. 1999 The geography of spermatophytic families and genera. Science Press, Beijing, China.

[ece32014-bib-0038] Manchester, S. R. , Z. D. Chen , A. M. Lu , and K. Uemura . 2009 Eastern Asian endemic seed plant genera and their paleogeographic history throughout the Northern Hemisphere. J. Syst. Evol. 47:1–42.

[ece32014-bib-0039] Michalakis, Y. , and L. Excoffier . 1996 A generic estimation of population subdivision using distances between alleles with special reference for microsatellite loci. Genetics 142:1061–1064. 8849912 10.1093/genetics/142.3.1061 PMC1207006

[ece32014-bib-0040] Ni, J. , X. Cao , F. Jeltsch , and U. Herzschuh . 2014 Biome distribution over the last 22,000 yr in China. Palaeogeogr. Palaeoclimatol. Palaeoecol. 409:33–47.

[ece32014-bib-0041] Nielsen, R. , and J. Wakeley . 2001 Distinguishing migration from isolation: a Markov chain Monte Carlo approach. Genetics 158:885–896. 11404349 10.1093/genetics/158.2.885 PMC1461674

[ece32014-bib-0042] O'Connell, L. M. , and K. Ritland . 2004 Somatic mutations at microsatellite loci in western redcedar (*Thuja plicata*: Cupressaceae). J. Hered. 95:172–176. 15073234 10.1093/jhered/esh024

[ece32014-bib-0043] Posada, D. 2008 jModelTest: phylogenetic model averaging. Mol. Biol. Evol. 25:1253–1256. 18397919 10.1093/molbev/msn083

[ece32014-bib-0044] Pritchard, J. K. , M. Stephens , and P. Donnelly . 2000 Inference of population structure using multilocus genotype data. Genetics 155:945–959. 10835412 10.1093/genetics/155.2.945 PMC1461096

[ece32014-bib-0045] Qi, X. S. , C. Chen , H. P. Comes , S. Sakaguchi , Y. H. Liu , N. Tanaka , et al. 2012 Molecular data and ecological niche modelling reveal a highly dynamic evolutionary history of the East Asian Tertiary relict *Cercidiphyllum* (Cercidiphyllaceae). New Phytol. 196:617–630. 22845876 10.1111/j.1469-8137.2012.04242.x

[ece32014-bib-0046] Qian, H. , and R. E. Ricklefs . 2000 Large‐scale processes and the Asian bias in species diversity of temperate plants. Nature 407:180–182. 11001054 10.1038/35025052

[ece32014-bib-0047] Qiu, Y. X. , B. C. Guan , C. X. Fu , and H. P. Comes . 2009a Did glacials and/or interglacials promote allopatric incipient speciation in East Asian temperate plants? Phylogeographic and coalescent analyses on refugial isolation and divergence in *Dysosma versipellis* . Mol. Phylogenet. Evol. 51:281–293. 19405195 10.1016/j.ympev.2009.01.016

[ece32014-bib-0048] Qiu, Y. X. , X. S. Qi , X. F. Jin , X. Y. Tao , C. X. Fu , A. Naiki , et al. 2009b Population genetic structure, phylogeography, and demographic history of *Platycrater arguta* (Hydrangeaceae) endemic to East China and South Japan, inferred from chloroplast DNA sequence variation. Taxon 58:1226–1241.

[ece32014-bib-0049] Qiu, Y. X. , Y. Sun , X. P. Zhang , J. Lee , C. X. Fu , and H. P. Comes . 2009c Molecular phylogeography of East Asian *Kirengeshoma* (Hydrangeaceae) in relation to Quaternary climate change and landbridge configurations. New Phytol. 183:480–495. 19496955 10.1111/j.1469-8137.2009.02876.x

[ece32014-bib-0050] Qiu, Y. X. , C. X. Fu , and H. P. Comes . 2011 Plant molecular phylogeography in China and adjacent regions: tracing the genetic imprints of Quaternary climate and environmental change in the world's most diverse temperate flora. Mol. Phylogenet. Evol. 59:225–244. 21292014 10.1016/j.ympev.2011.01.012

[ece32014-bib-0051] Rambaut, A. , and A. J. Drummond . 2009 Tracer v1.5. http://beast.bio.ed.ac.uk/Tracer

[ece32014-bib-0052] Ranius, T. , M. Niklasson , and N. Berg . 2009 Development of tree hollows in pedunculate oak (*Quercus robur*). For. Ecol. Manage. 257:303–310.

[ece32014-bib-0053] Rice, W. R. 1989 Analyzing tables of statistical tests. Evolution 43:223–225. 10.1111/j.1558-5646.1989.tb04220.x 28568501

[ece32014-bib-0054] Ronquist, F. , and J. P. Huelsenbeck . 2003 MrBayes 3: Bayesian phylogenetic inference under mixed models. Bioinformatics 19:1572–1574. 12912839 10.1093/bioinformatics/btg180

[ece32014-bib-0055] Royer, D. L. , L. J. Hickey , and S. L. Wing . 2003 Ecological conservatism in the “living fossil” *Ginkgo* . Paleobiology 29:84–104.

[ece32014-bib-0056] Sakaguchi, S. , Y. Qiu , Y. Liu , X. Qi , S. Kim , J. Han , et al. 2012 Climate oscillation during the Quaternary associated with landscape heterogeneity promoted allopatric lineage divergence of a temperate tree *Kalopanax septemlobus* (Araliaceae) in East Asia. Mol. Ecol. 21:3823–3838. 22646502 10.1111/j.1365-294X.2012.05652.x

[ece32014-bib-0057] Schneider, S. , and L. Excoffier . 1999 Estimation of past demographic parameters from the distribution of pairwise differences when the mutation rates vary among sites: application to human mitochondrial DNA. Genetics 152:1079–1089. 10388826 10.1093/genetics/152.3.1079 PMC1460660

[ece32014-bib-0058] Shen, L. , X. Y. Chen , X. Zhang , Y. Y. Li , C. X. Fu , and Y. X. Qiu . 2005 Genetic variation of *Ginkgo biloba* L. (Ginkgoaceae) based on cpDNA PCR‐RFLPs: inference of glacial refugia. Heredity 94:396–401. 15536482 10.1038/sj.hdy.6800616

[ece32014-bib-0059] Slatkin, M. 1987 Gene flow and the geographic structure of natural populations. Science 236:787–792. 3576198 10.1126/science.3576198

[ece32014-bib-0060] Tajima, F. 1989 Statistical method for testing the neutral mutation hypothesis by DNA polymorphism. Genetics 123:585–595. 2513255 10.1093/genetics/123.3.585 PMC1203831

[ece32014-bib-0061] Tamura, K. , and M. Nei . 1993 Estimation of the number of nucleotide substitutions in the control region of mitochondrial DNA in humans and chimpanzees. Mol. Biol. Evol. 10:512–526. 8336541 10.1093/oxfordjournals.molbev.a040023

[ece32014-bib-0062] Tang, C. Q. , Y. Yang , M. Ohsawa , S. Yi , A. Momohara , W. Su , et al. 2012 Evidence for the persistence of wild *Ginkgo biloba* (Ginkgoaceae) populations in the Dalou Mountains, southwestern China. Am. J. Bot. 99:1408–1414. 22847538 10.3732/ajb.1200168

[ece32014-bib-0063] Wang, J. , P. Gao , M. Kang , A. J. Lowe , and H. Huang . 2009 Refugia within refugia: the case study of a canopy tree (*Eurycorymbus cavaleriei*) in subtropical China. J. Biogeogr. 36:2156–2164.

[ece32014-bib-0064] Willyard, A. , J. Syring , D. Gernandt , A. Liston , and R. Cronn . 2007 Fossil calibration of molecular divergence infers a moderate mutation rate and recent radiations for *Pinus* . Mol. Biol. Evol. 24:90–101. 16997907 10.1093/molbev/msl131

[ece32014-bib-0065] Wu, C. , S. Chaw , and Y. Huang . 2013 Chloroplast phylogenomics indicates that *Ginkgo biloba* is sister to cycads. Genome Biol. Evol. 5:243–254. 23315384 10.1093/gbe/evt001 PMC3595029

[ece32014-bib-0066] Yan, X. F. , C. L. Lian , and T. Hogetsu . 2006 Development of microsatellite markers in ginkgo (*Ginkgo biloba* L.). Mol. Ecol. Notes 6:301–302.

[ece32014-bib-0067] Yan, X. L. , Y. Y. Chen , B. C. Guan , and C. X. Fu . 2009 Eleven novel microsatellite markers developed from the living fossil *Ginkgo biloba* (Ginkgoaceae). Conserv. Genet. 10:1277–1279.

[ece32014-bib-0068] Yi, C. , Z. Cui , and H. Xiong . 2005 Numerical periods of quaternary glaciations in China. Quat. Sci. 25:609–619.

