# Supplementary material for: Incongruent range dynamics between co‐occurring Asian temperate tree species facilitated by life history traits
Source: Ecol Evol. 2016 Mar 6;6(8):2346–58. doi: 10.1002/ece3.2014 (PMC4782244; doi:10.1002/ece3.2014)
Supplement: Supplementary file 1 — Appendix S1. Sampling information, GenBank accession numbers, population diversity of nSSRs and cpDNA haplotypes, tests of IM nested models, neutrality tests, and likelihood values of the cluster number in Bayesian assignment analyses. Table S1. Details of sample locations, voucher number, latitude, longitude and altitude in 25 G. biloba populations. Table S2. GenBank accession numbers of cpDNA variants. Table S3. Locus, repeat motifs, size ranges, number of alleles (N A), observed (H O) and expected heterozygosity (H E) for fourteen microsatellite loci analyzed in 25 populations of G. biloba. Table S4. Chloroplast DNA sequence polymorphisms detected in the three regions of Ginkgo biloba identify 13 haplotypes (H1–H13). Table S5. Plastid and nuclear diversity in 25 G. biloba populations sampled across its complete natural range in China. Table S6. Tests of nested models for the WEST versus EAST group for the three cpDNA loci and 14 nSSR loci data set. Table S7. Summary of neutrality tests and mismatch distribution parameters for groups WEST and EAST of G. biloba. Estimates of mismatch distribution were obtained under models of spatial expansion using ARLEQUIN. Fig. S1 Likelihood values across multiple values of the cluster number in Bayesian assignment analyses, K (1–10) using Evanno's methods (Evanno et al. 2005). [file ECE3-6-2346-s001.docx]

**SUPPORTING INFORMATION**

**Incongruent range dynamics between co-occurring Asian temperate tree species facilitated by life history traits**

Yun-Peng Zhao, Xiao-Ling Yan, Graham Muir, Qiong-Yan Dai, Marcus A. Koch, Cheng-Xin Fu

**Appendix S1** Sampling information, genbank accession numbers, population diversity of nSSRs and cpDNA haplotypes, tests of IM nested models, neutral tests, and likelihood values of the cluster number in Bayesian assignment analyses.

**Table S1.** Details of sample locations, voucher number, latitude, longitude and altitude in 25 *G. biloba* populations. Voucher specimens are all deposited in the Herbarium of Zhejiang University (HZU).

| Population code | Locations | Voucher number | Longitude (E) | Latitude (N) | Altitude (m) |
| --- | --- | --- | --- | --- | --- |
| 1. PX | Panxian, Guizhou | QL2002PX104 | 104°64′ | 25°81′ | > 1500 |
| 2. SP | Shanping, Guizhou | GW2004SP183 | 106°48′ | 26°15′ | 1500 |
| 3. WC | Wuchuan, Guizhou | GD2001WC011 | 107°53′ | 28°26′ | 550–1200 |
| 4. JF | Mt. Jinfo, Chongqing | FG2003JF135 | 107°15′ | 28°56′ | 800–1300 |
| 5. QC | Mt. Qingcheng, Sichuan | YX2008QC005 | 103°33′ | 30°54′ | > 1100 |
| 6. ES | Enshi, Hubei | GW2004ES173 | 109°45′ | 30°25′ | 1025 |
| 7. HP | Mt. Huping, Hunan | YX2008HP009 | 110°39′ | 29°56′ | 830–1224 |
| 8. SN | Shennongjia, Hubei | YX2008SN001 | 110°34′ | 31°19′ | 800–1100 |
| 9. HX | Huixian, Gansu | YX2008HX028 | 106°10′ | 33°44′ | 826 |
| 10. XA^*^ | Xi'an, Shanxi | YX2008XA002 | 108°54′ | 34°15′ | 400 |
| 11. SX^*^ | Songxian, Henan | GY2002SX067 | 112°42′ | 31°09′ | 540–870 |
| 12. DH | Mt. Dahong, Hubei | GY2002DH021 | 113°30′ | 31°20′ | 200–700 |
| 13. XX | Xinxian, Henan | YX2008XX001 | 114°45′ | 31°45′ | 115 |
| 14. JZ | Jinzhai, Anhui | YX2008JZ004 | 115°27′ | 31°21′ | 300 |
| 15. YX^*^ | Yongxiu, Jiangxi | YX2008YX002 | 114°22′ | 26°59′ | 500 |
| 16. HS^*^ | Hengshan, Hunan | YX2008HS001 | 112°73′ | 27°24′ | 800 |
| 17. GX^*^ | Lingchun, Guangxi | GD2001GX001 | 110°30′ | 25°24′ | 200 |
| 18. SG^*^ | Shaoguan, Guangdong | YX2008SG001 | 114°39′ | 25°08′ | 170 |
| 19. WY^*^ | Mt. Wuyi, Fujian | GY2001WY094 | 118°00′ | 27°06′ | > 1000 |
| 20. TM | Mt. Tianmu, Zhejiang | CC2006TM301 | 119°26′ | 30°19′ | 380–1105 |
| 21. CX^*^ | Changxing, Zhejiang | YG2005CX037 | 119°42′ | 31°01′ | 200 |
| 22. JR^*^ | Jurong, Jiangsu | ZY2009JR001 | 119°30′ | 32°42 | 252 |
| 23. TX^*^ | Taixing, jiangsu | GY2001TX057 | 120°01′ | 32°12′ | <10 |
| 24. TC^*^ | Tancheng, Shandong | GY2001TC080 | 118°16′ | 34°39′ | 200 |
| 25. ZH^*^ | Zunhua, Hebei | YX2008ZH001 | 118°00′ | 40°17′ | 220 |

Asterisks indicate cultivated populations.

**Table S2.** GenBank accession numbers of cpDNA variants. Haplotype names correspond to samples in Table S5.

| haplotype | *trn*K | *trn*S-*trn*G | *atp*H-*atp*I |
| --- | --- | --- | --- |
| H1 | EF468637 | EF468641 | KF220584 |
| H2 | EF468638 | EF468641 | KF220584 |
| H3 | EF468637 | EF468641 | KF220585 |
| H4 | EF468635 | EF468642 | KF220585 |
| H5 | EF468635 | EF468642 | KF220584 |
| H6 | EF468632 | EF468642 | KF220585 |
| H7 | EF468633 | EF468642 | KF220585 |
| H8 | EF468632 | EF468641 | KF220584 |
| H9 | EF468635 | EF468641 | KF220585 |
| H10 | EF468637 | EF468641 | KF220586 |
| H11 | EF468640 | EF468641 | KF220586 |
| H12 | EF468640 | EF468641 | KF220584 |
| H13 | KF220583 | EF468641 | KF220586 |

**Table S3.** Locus, repeat motifs, size ranges, number of alleles (*N*_A_), observed (*H*_O_) and expected heterozygosity (*H*_E_) for fourteen microsatellite loci analyzed in 25 populations of *G. biloba*.

| Reference | Locus | Repeat motis | Ta (°C) | Size range | *N_A_* | *H_O_* | *H_E_* |
| --- | --- | --- | --- | --- | --- | --- | --- |
| Yan et al. (2006) | Ging01 | (TC)_7_(TA)_8_ | 53 | 305–347 | 17 | 0.461 | 0.667 |
|  | Ging04 | (GT)_7_AA(GA)_9_ | 54 | 147–151 | 3 | 0.69 | 0.573 |
|  | Ging05 | (TG)_7_(AG)_9_ | 54 | 203–265 | 29 | 0.777 | 0.928 |
|  | Ging06 | (GA)_14_ | 54 | 217–279 | 29 | 0.87 | 0.946 |
|  | Ging11 | (TG)_8_(AG)_9_ | 54 | 204–244 | 17 | 0.791 | 0.85 |
| Yan et al. (2009) | Gb43 | (GT)_16_ | 58 | 91–109 | 10 | 0.728 | 0.742 |
|  | Gb44 | (AC)_12_ | 53 | 371–413 | 12 | 0.446 | 0.74 |
|  | Gb48 | (TC)_16_(AC)_23_ | 60 | 224–300 | 28 | 0.731 | 0.764 |
|  | Gb54 | (TC)_13_(AC)_17_ | 58 | 79–123 | 20 | 0.748 | 0.84 |
|  | Gb57 | (TC)_16_(AC)_12_AT(AC)_5_ | 53 | 157–217 | 29 | 0.751 | 0.882 |
|  | Gb60 | (TG)_10_(AG)_16_ | 55 | 233–273 | 20 | 0.717 | 0.882 |
|  | Gb61 | (GT)_18_ | 62 | 361–423 | 29 | 0.804 | 0.932 |
|  | Gb64 | (TG) _13_ | 58 | 151–173 | 7 | 0.533 | 0.707 |
|  | Gb65 | (TC)_8_(AC)_15_ | 58 | 127–149 | 11 | 0.748 | 0.791 |
|  | Mean |  |  |  | 18.6 | 0.699 | 0.803 |

Yan XF, Lian CL, Hogetsu T (2006) Development of microsatellite markers in ginkgo (*Ginkgo biloba* L.). *Molecular Ecology Notes* **6**, 301–302.

Yan XL, Chen YY, Guan BC, Fu CX (2009) Eleven novel microsatellite markers developed from the living fossil *Ginkgo biloba* (Ginkgoaceae). *Conservation Genetics* **10**, 1277–1279.

**Table S4.** Chloroplast DNA sequence polymorphisms detected in the three regions of *Ginkgo biloba* identify 13 haplotypes (H1–H13). The 2118 bp aligned sequences of 354 individuals from 25 populations present 11 non-repeat polymorphic characters which include nine single nucleotide substitutions, one inversion and one indel (22 bp). The *trn*K intron is the most variable (nine polymorphisms in 1040 aligned positions; 0.86%), followed by *atp*H–*atp*I IGS (2/479; 0.42%) and *trn*S–*trn*G IGS (1/599; 0.17%).

| Haplotype | *trn*K　intron | | | | | | | |  | *trn*S–G |  | *atp*H–*atp*I | |
| --- | --- | --- | --- | --- | --- | --- | --- | --- | --- | --- | --- | --- | --- |
|  | 88 | 109 | 148 | 204 | 770 | 884 | 890 | 901 |  | 1167 |  | 1690 | 1720 |
| H1 | T | A | A | 2^a^ | T | C | T | G |  | G |  | 1^c^ | C |
| H2 | . | . | . | 2^a^ | . | . | C | . |  | . |  | 1^c^ | . |
| H3 | . | . | . | 2^a^ | . | . | . | . |  | . |  | 0 | . |
| H4 | . | . | . | 2^a^ | C | T | . | . |  | A |  | 0 | . |
| H5 | . | . | . | 2^a^ | C | T | . | . |  | A |  | 1^c^ | . |
| H6 | . | . | . | 2^b^ | C | T | . | . |  | A |  | 0 | . |
| H7 | G | T | T | 2^b^ | C | T | . | . |  | A |  | 0 | . |
| H8 | . | . | . | 2^b^ | C | T | . | . |  | . |  | 1^c^ | . |
| H9 | . | . | . | 2^a^ | C | T | . | . |  | . |  | 0 | . |
| H10 | . | . | . | 2^a^ | . | . | . | . |  | . |  | 1^c^ | A |
| H11 | . | . | . | 2^b^ | . | . | . | . |  | . |  | 1^c^ | A |
| H12 | . | . | . | 2^b^ | . | . | . | . |  | . |  | 1^c^ | . |
| H13 | . | . | . | 2^a^ | . | . | . | A |  | . |  | 1^c^ | A |

All sequences are compared to the reference haplotype H1. Numbers '0/1' in the sequences indicate absence/presence of length polymorphisms and '2' presence of an inversion, whereby superscripts identify corresponding character states.

^a^ TC; ^b^ GA; ^c^ ATTACATGTCCTAATATACGCA

**Table S5.** Plastid and nuclear diversity in 25 *G. biloba* populations sampled across its complete natural range in China.

| Population code | Location | cpDNA | | | |  | nSSR | | | | | |
| --- | --- | --- | --- | --- | --- | --- | --- | --- | --- | --- | --- | --- |
|  |  | *n* | *h* | * *× 10^3^ | Haplotypes (Number) |  | *n* | *N*_A_ | *A*_R_ | *RAR* | *H*_E_ | *H*_O_ |
| 1. PX | Panxian, Guizhou | 10 | 0.200 | 0.283 | H1(9), H5(1) |  | 14 | 68 | 4.39 | 0.132 | 0.706 | 0.709 |
| 2. SP | Shaping, Guizhou | 10 | 0.000 | 0.000 | H1(10) |  | 13 | 67 | 4.15 | 0.298 | 0.642 | 0.676 |
| 3. WC | Wuchuan, Guizhou | 21 | 0.701 | 1.112 | H1(13), H3(1), H4(5), H5(1), H8(1) |  | 102 | 165 | 5.91 | 0.564 | 0.741 | 0.645 |
| 4. JF | Mt. Jinfo, Chongqing | 16 | 0.572 | 1.369 | H1(4), H3(2), H6(9), H7(1) |  | 61 | 128 | 4.94 | 0.547 | 0.690 | 0.679 |
| 5. QC | Mt. Qingcheng, Sichuan | 10 | 0.183 | 0.343 | H1(9), H4(1) |  | 11 | 70 | 4.55 | 0.285 | 0.654 | 0.734 |
| 6. ES | Badong, Hubei | 10 | 0.600 | 0.976 | H1(6), H4(3), H5(1) |  | 17 | 94 | 5.25 | 0.457 | 0.690 | 0.685 |
| 7. HP | Mt. Huping, Hunan | 9 | 0.500 | 0.472 | H4(3), H6(6) |  | 9 | 79 | 5.64 | 0.329 | 0.727 | 0.625 |
| 8. SN | Shennongjia, Hubei | 7 | 0.667 | 1.439 | H1(2), H4(1), H6(4) |  | 8 | 88 | 5.98 | 0.420 | 0.735 | 0.718 |
| 9. HX | Huixian, Gansu | 9 | 0.389 | 0.550 | H1(7), H9(2) |  | 20 | 104 | 5.71 | 0.365 | 0.758 | 0.671 |
| 10. XA^*^ | Xi'an, Shaanxi | 10 | 0.200 | 0.283 | H1(9), H11(1) |  | 11 | 92 | 6.11 | 0.207 | 0.826 | 0.734 |
| **Western** |  | 112 | 0.603 | 1.190 | 9: H1(69), H3(3), H4(13), H5(3), H6(19), H7(1), H8(1), H9(2), H11(1) |  | 266 | 96 | 5.26 | 0.360 | 0.717 | 0.688 |
| 11. SX^*^ | Songxian, Henan | 10 | 0.000 | 0.000 | H1(10) |  | 10 | 60 | 3.99 | 0.317 | 0.610 | 0.607 |
| 12. DH | Mt. Dahong, Hubei | 23 | 0.443 | 0.210 | H1(16), H3(7) |  | 24 | 117 | 6.12 | 0.350 | 0.777 | 0.768 |
| 13. XX | Xinxian, Henan | 12 | 0.530 | 1.020 | H1(5), H4(7) |  | 17 | 102 | 5.72 | 0.431 | 0.757 | 0.798 |
| 14. JZ | Jinzhai, Anhui | 15 | 0.000 | 0.000 | H1(15) |  | 20 | 114 | 5.99 | 0.403 | 0.740 | 0.636 |
| 15. YX^*^ | Yongxiu, Jiangxi | 8 | 0.000 | 0.000 | H1(8) |  | 10 | 80 | 5.29 | 0.338 | 0.722 | 0.693 |
| 16. HS^*^ | Hengshan, Hunan | 9 | 0.000 | 0.000 | H1(9) |  | 10 | 49 | 3.33 | 0.224 | 0.619 | 0.762 |
| 17. GX^*^ | Lingchuan, Guangxi | 9 | 0.389 | 0.551 | H1(7), H5(2) |  | 18 | 87 | 5.13 | 0.287 | 0.720 | 0.726 |
| 18. SG^*^ | Nanxiong, Guangdong | 11 | 0.000 | 0.000 | H1(11) |  | 13 | 78 | 4.90 | 0.358 | 0.668 | 0.615 |
| **Central** |  | 97 | 0.295 | 0.360 | 4: H1(81), H3(7), H4(7), H5(2) |  | 122 | 86 | 5.06 | 0.338 | 0.702 | 0.700 |
| 19. WY^*^ | Mt. Wuyi, Fujian | 7 | 0.000 | 0.000 | H1(7) |  | 11 | 83 | 5.14 | 0.421 | 0.759 | 0.695 |
| 20. TM | Mt. Tianmu, Zhejiang | 64 | 0.695 | 0.732 | H1(42), H10(3), H11(7), H12(5), H13(7) |  | 266 | 211 | 6.57 | 0.654 | 0.788 | 0.738 |
| 21. CX^*^ | Changxing, Zhejiang | 37 | 0.052 | 0.026 | H1(36), H2 (1) |  | 38 | 132 | 5.90 | 0.432 | 0.768 | 0.708 |
| 22. TX^*^ | Taixing, Jiangsu | 8 | 0.000 | 0.000 | H1(8) |  | 13 | 66 | 4.15 | 0.227 | 0.673 | 0.753 |
| 23. JR^*^ | Jurong, Jiangsu | 8 | 0.000 | 0.000 | H1(8) |  | 8 | 83 | 5.67 | 0.421 | 0.771 | 0.768 |
| 24. TC^*^ | Tancheng, Shandong | 10 | 0.000 | 0.000 | H1(10) |  | 14 | 73 | 4.17 | 0.383 | 0.629 | 0.832 |
| 25. ZH^*^ | Zunhua, Hebei | 11 | 0.000 | 0.000 | H1(11) |  | 12 | 55 | 3.68 | 0.145 | 0.621 | 0.726 |
| **Eastern** |  | 145 | 0.288 | 0.290 | 6: H1(122), H2(1), H10(3), H11(7), H12(5), H13(7) |  | 362 | 100 | 5.04 | 0.383 | 0.716 | 0.746 |
| **All** |  | 354 | 0.439±0.034 | 0.740±0.310 | 13: H1(272), H2(1), H3(10), H4(20), H5(5), H6(19), H7(1), H8(1), H9(2), H10(3), H11(8), H12(5), H13(7) |  |  | 93 | 5.10 | 0.358 | 0.710 | 0.705 |

Asterisks indicate presumably cultivated populations. Cytoplast parameters: sample size (*n*), haplotype diversity (*h*), nucleotide diversity (*π* ) and haplotypes number. Nuclear parameters: sample size (*n*), the number of alleles per locus (*N_A_*), allelic richness (*A_R_*), rare allelic richness (*RAR*), the number of private alleles (N*_PA_*), allelic diversity (*H*_E_), heterozygosity (*H*_O_).

**Table S6.** Tests of nested models for the WEST vs EAST group for the three cpDNA loci and 14 nSSR loci data set

| Model parameters | log (*P*) | *q_1_* | *q_2_* | *q_A_* | *m_1_* | *m_2_* | *df* | -2LLR | *P* |
| --- | --- | --- | --- | --- | --- | --- | --- | --- | --- |
| Full model | -4.034 | 0.8652 | 1.111 | 63.4839 | 0 | 5.7888 | - | - |  |
| *m*_1_ = *m*_2_ | -4.979 | 0.8603 | 1.6516 | 52.1561 | 2.3659 | [2.3659] | 1 | 1.889 | 0.046824 |
| *m*_1_ = 0 | -4.034 | 0.8652 | 1.111 | 63.4839 | [0.00000] | 5.7887 | 1* | 0 | 1 |
| *m*_2_ = 0 | -6.088 | 0.7381 | 2.8316 | 88.6844 | 5.1474 | [0.00000] | 1* | 4.107 | 0.023424 |
| *m*_1_ = *m*_2_ = 0 | -435 | 1.0999 | 2.3365 | 32.439 | [0.00000] | [0.00000] | 2* | 862 | 1.42E-58 |
| *θ*_1_ = *θ*_2_ | -5.08 | 1.5829 | [1.5829] | 78.5571 | 0 | 4.1754 | 1 | 2.092 | 0.037367 |
| *θ*_1_ = *θ*_2_, *m*_1_ = *m*_2_ | -9.86 | 1.6414 | [1.6414] | 78.0885 | 1.4636 | [1.4636] | 2 | 11.65 | 0.003603 |
| *θ*_1_ = *θ*_2_, *m*_1_ = 0 | -5.08 | 1.5829 | [1.5829] | 78.5572 | [0.00000] | 4.1754 | 2* | 2.092 | 0.037367 |
| *θ*_1_ = *θ*_2_, *m*_2_ = 0 | -185.9 | 0.937 | [0.9370] | 58.2583 | 2.2428 | [0.00000] | 2* | 363.7 | 9.70E-47 |
| *θ*_1_ = *θ*_2_, *m*_1_ = *m*_2_ = 0 | -512 | 1.664 | [1.6640] | 32.439 | [0.00000] | [0.00000] | 3* | 1016 | 0 |
| *θ*_1_ = *θ*_A_ | -699 | 9.2488 | 3.0913 | [9.2488] | 0.1324 | 1.6231 | 1 | 1390 | 0 |
| *θ*_1_ = *θ*_A_, *m*_1_ = *m*_2_ | -759.1 | 9.2488 | 3.0913 | [9.2488] | 0.8434 | [0.8434] | 2 | 1510 | 0 |
| *θ*_1_ = *θ*_A_, *m*_1_ = 0 | -843 | 9.2488 | 3.0913 | [9.2488] | [0.00000] | 1.6231 | 2* | 1678 | 0 |
| *θ*_1_ = *θ*_A_, *m*_2_ = 0 | -1201 | 14.6064 | 3.3021 | [14.6064] | 3.3495 | [0.00000] | 2* | 2393 | 0 |
| *θ*_1_ = *θ*_A_, *m*_1_ = *m*_2_ =0 | -1556 | 12.4746 | 2.3365 | [12.4746] | [0.00000] | [0.00000] | 3* | 3104 | 0 |
| *θ*_1_ = *θ*_A_ | -421.9 | 0.8492 | 8.2732 | [8.2732] | 4.2236 | 0.6499 | 1 | 835.6 | 1.25E-57 |
| *θ*_1_ = *θ*_A_, *m*_1_ = *m*_2_ | -441.4 | 1.6019 | 12.4061 | [12.4061] | 1.1151 | [1.1151] | 2 | 874.8 | 5.39E-58 |
| *θ*_1_ = *θ*_A_, *m*_1_ = 0 | -541.6 | 2.1359 | 12.6404 | [12.6404] | [0.00000] | 1.973 | 2* | 1075 | 0 |
| *θ*_1_ = *θ*_A_, *m*_2_ = 0 | -637.3 | 0.8073 | 12.2659 | [12.2659] | 3.0908 | [0.00000] | 2* | 1266 | 0 |
| *θ*_1_ = *θ*_A_, *m*_1_ = *m*_2_ =0 | -1074 | 1.0999 | 14.514 | [14.5140] | [0.00000] | [0.00000] | 3* | 2140 | 0 |
| *θ*_1_ = *θ*_2_ = *θ*_A_ | -874.5 | 6.9757 | [6.9757] | [6.9757] | 0.1324 | 1.6231 | 2 | 1741 | 0 |
| *θ*_1_ = *θ*_2_ = *θ*_A_, *m*_1_ = *m*_2_ | -934.7 | 6.9757 | [6.9757] | [6.9757] | 0.8434 | [0.8434] | 3 | 1861 | 0 |
| *θ*_1_ = *θ*_2_ = *θ*_A_, *m*_1_ = 0 | -1019 | 6.9757 | [6.9757] | [6.9757] | [0.00000] | 1.6231 | 3* | 2029 | 0 |
| *θ*_1_ = *θ*_2_ = *θ*_A_, *m*_2_ = 0 | -1484 | 7.7462 | [7.7462] | [7.7462] | 2.5304 | [0.00000] | 3* | 2961 | 0 |
| *θ*_1_ = *θ*_2_ = *θ*_A_, *m*_1_ = *m*_2_ =0 | -1915 | 8.9443 | [8.9443] | [8.9443] | [0.00000] | [0.00000] | 4* | 3822 | 0 |

log (*P*): posterior density of the model parameters given the data.

LLR: log-likelihood ratio of the full model and the nested model.

*P*: probability of achieving the test statistic (-2LLR) by chance under the null model.

*expected distribution of -2LLR is a mixture.

**Table S7.** Summary of neutrality tests and mismatch distribution parameters for groups WEST and EAST of *G. biloba*. Estimates of mismatch distribution were obtained under models of spatial expansion using ARLEQUIN.

| Group | Tajima’s *D* | *P* | Fu’s *F*_S_ | *P* | SSD | *P* | *H*_Rag_ | *P* |
| --- | --- | --- | --- | --- | --- | --- | --- | --- |
| WEST | 0.484 | 0.750 | 1.674 | 0.802 | 0.414 | 0.000 | 0.178 | 0.998 |
| EAST | 0.936 | 0.864 | 0.676 | 0.643 | 0.074 | 0.091 | 0.194 | 0.065 |


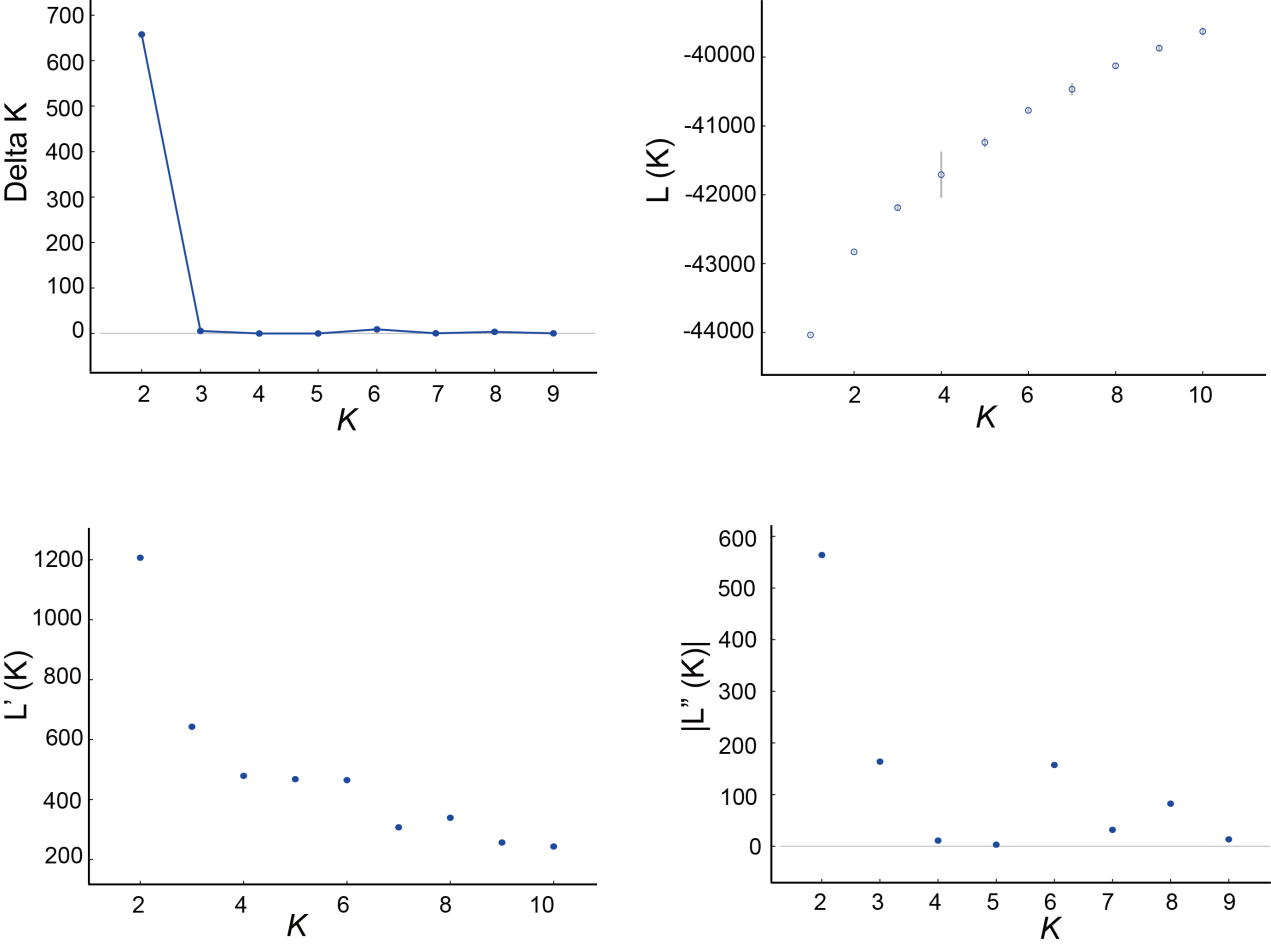


**Fig. S1** Likelihood values across multiple values of the cluster number in Bayesian assignment analyses, *K* (1 to 10) using Evanno’s methods (Evanno *et al.*, 2005). The *K* value with the greatest log-likelihood value suggests the number of genetic groups that best fit the data.
